# No evidence for social immunity in co-founding queen associations

**DOI:** 10.1038/s41598-017-16368-4

**Published:** 2017-11-24

**Authors:** Timothée Brütsch, Amaury Avril, Michel Chapuisat

**Affiliations:** Department of Ecology and Evolution, Biophore, UNIL-Sorge, University of Lausanne, 1015 Lausanne, Switzerland

## Abstract

Ant queens often associate to found new colonies, yet the benefits of this behaviour remain unclear. A major hypothesis is that queens founding in groups are protected by social immunity and can better resist disease than solitary queens, due to mutual grooming, sharing of antimicrobials, or higher genetic diversity among their workers. We tested this hypothesis by manipulating the number of queens in incipient colonies of *Lasius niger* and measuring their resistance to the fungal entomopathogen *Metarhizium brunneum*. We found no evidence for social immunity in associations of founding queens. First, co-founding queens engaged in self-grooming, but performed very little allo-grooming or trophallaxis. Second, co-founding queens did not exhibit higher pathogen resistance than solitary queens, and their respective workers did not differ in disease resistance. Finally, queens founding in groups increased their investment in a component of individual immunity, as expected if they do not benefit from social immunity but respond to a higher risk of disease. Overall, our results provide no evidence that joint colony founding by *L*. *niger* queens increases their ability to resist fungal pathogens.

## Introduction

In social insects, founding novel colonies is a risky enterprise. Many ant species produce massive numbers of queens that fly away from their natal nest, mate, and seek to establish colonies independently^1,2^. Incipient colonies are vulnerable to predation, competition and disease – their mortality has been estimated to be as high as 95%^3^. Ant queens that are able to found colonies independently often associate with other queens, a mode of foundation called pleometrosis^2,4^. These associations are transient: after the first workers emerge, the queens fight to death, until only one remains^2^. Hence, joint colony founding is a gamble for queens, with maximal costs to losers and large benefits to the winner^4^.

A major potential benefit of joint colony founding by ant queens is increased disease resistance, which might stem from various mechanisms conferring social immunity^5,6^. Queens in group may benefit from mutual grooming^7^. They may also share antimicrobial substances from the metapleural glands^8^, the venom gland^9^, or in trophallactic fluids^10^. Finally, queens in associations will produce groups of workers that are initially genetically more diverse, until queen fight^2,11^, and which might therefore better resist pathogens^12,13^.

The benefits from social immunity might be crucial during claustral colony founding, when queens rely on their body reserves and are under strong energetic stress^3^. If joint colony founding confers social immunity, the queens may decrease their investment in energetically costly individual immunity^14^. Such a trade-off has been documented in wood ants, who showed lower activation of their immune system when antimicrobial resin was present in their nests^15^. Conversely, in absence of social immunity, queens in groups are likely to increase individual immunity in order to respond to higher disease risk^16–18^. Such density-dependent prophylaxis has been documented in locusts, thrips and Lepidoptera^19–21^.

So far, evidence for social immunity in associations of co-founding ant queens have remained elusive. In *Lasius niger*, queens founding in pairs did not engage in allo-grooming and did not show higher survival than solitary queens when exposed to the fungal pathogen *Metarhizium pingshaense* ^5^. In *Formica selysi*, the presence of a fungal pathogen in the nest did not incite queens to associate, as would be expected if joint colony founding would increase their ability to resist the pathogen^6^.

Here, we investigated if ant queens that found colonies in associations benefit from social immunity and modulate their individual immunity. We established experimental incipient colonies of *L*. *niger* with one, two or four queens, respectively, as occurs regularly in nature^2^. We tested the resistance of queens to the common soil entomopathogen *Metarhizium brunneum* and recorded their grooming behaviour. Finally, we complemented previous studies^5,6^ by monitoring three components of the queens’ individual immune system and testing the fungal resistance of their workers. If ant queens in associations profit from social immunity, we predict that co-founding queens will (i) show higher resistance to the fungal pathogen than solitary queens, (ii) engage in allo-grooming, (iii) decrease their investment in individual immunity, and (iv) produce groups of workers that are genetically more diverse and therefore more resistant to the fungal pathogen.

## Results

### Queen behaviour

Queens of *L*. *niger* in founding associations had very few social interactions. After being exposed to spores of the fungal pathogen *M*. *brunneum*, they did not increase allo-grooming. Across all experimental nests with two or four queens (45 nests in each category), we recorded only 12 occurrences of allo-grooming, out of a total of 17004 five-second scans (0.07%). Nine of these allo-grooming events occurred in the first week of observation, before any immune challenge. One occurred in the controls (cuticle puncture only), and two after the fungal challenge (cuticle puncture + exposure to *M*. *brunneum*). A single occurrence of trophallaxis was observed, after exposure to the pathogen.

**Figure 1:**
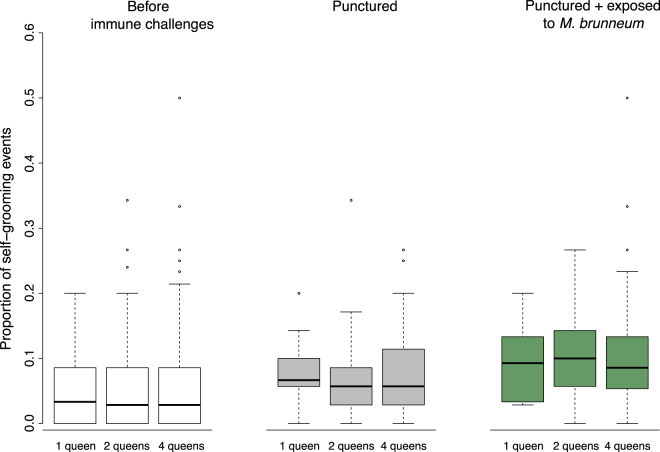
Proportion of self-grooming events, calculated as the number of occurrences of self-grooming divided by the total number of scans per queen alive. Boxplots show the median proportion of self-grooming events, as well as the upper and lower quartiles. The whiskers encompass 1.5 times the interquartile range. The proportion of self-grooming did not vary with the number of queens in founding associations (1, 2 or 4 queens), but increased after both types of immune challenges (punctured and exposed to *M. brunneum*; see Material and Methods for details).

In contrast to allo-grooming, self-grooming was frequent and increased after the immune challenges (Fig. 1). Across the 135 experimental nests (45 with one, two and four queens, respectively), we recorded 998 occurrences of self-grooming, out of a total of 19769 scans (5%). The proportion of self-grooming events did not vary with the number of queens in the incipient colony (Fig. 1; before immune challenges: *F* = 0.15, df = 2, *P* = 0.86; after cuticle puncture: *F = *0.19, df = 2, *P = *0.83). The proportion of self-grooming events increased significantly after cuticle puncture (Fig. 1; *F = *246.3, df = 1, *P < *0.0001) and further increased after exposure to fungal spores (Fig. 1; *F = *14.3, df = 1, *P = *0.0003).

### Queen survival

**Figure 2:**
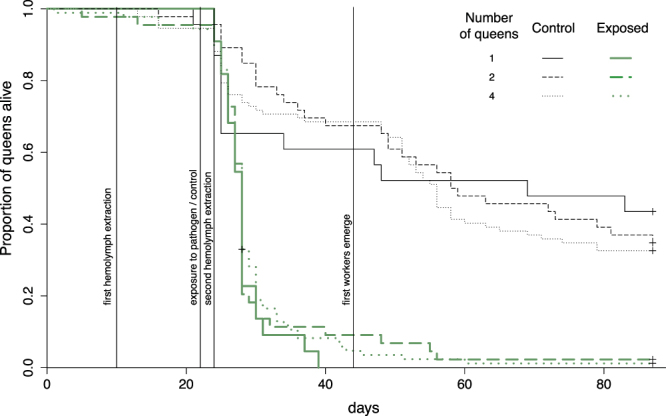
Survival of queens, in function of the number of queens in founding associations (1, 2 or 4 queens) and exposure to spores of M. *brunneum* (control versus exposed).

Over the entire time-course of the experiment, the fungal pathogen caused a strong and significant mortality to the queens (Fig. 2; χ^2^ = 117.9, df = 1, *P* < 0.0001). The number of queens in founding associations did not influence queen survival (Fig. 2; χ^2^ = 0.3, df = 2, *P* = 0.87). Queens that jointly founded incipient colonies did not show higher resistance to the pathogen than solitary queens (Fig. 2; no significant interaction between queen number and pathogen exposure, χ^2^ = 0.8, df = 1, *P* = 0.69). The pattern of queen mortality was very similar when restricting the analysis until the first workers emerged (Fig. 2; effect of pathogen exposure: χ^2^ = 116.9, df = 1, *P* < 0.0001; queen number: χ^2^ = 0.93, df = 2, *P* = 0.63; interaction: χ^2^ = 0.31, df = 2, *P* = 0.86).

### Worker survival

Most (85.5%) of the control nests (no fungal exposure) produced workers, which emerged between day 43 and 51 (average 45). In contrast, only one of the nests exposed to the fungal pathogen managed to produce a single worker. In control nests with queens alive at the end of the experiment, the total number of workers *per* nest increased with the number of queens (Mean ± SE: 14.6 ± 2.1, 18.6 ± 2.8 and 26.3 ± 3.1 workers *per* nest with 1, 2 and 4 founding queens, respectively; Kruskall-Wallis rank sum test: χ^2^ = 7.2, df = 2, *P* = 0.027). However, the number of workers *per* nest did not increase linearly with queen number, because the average *per capita* productivity of queens decreased in nests with more queens (14.6 ± 2.1, 9.3 ± 1.4 and 6.6 ± 0.79 workers *per* queen in nests with 1, 2 and 4 founding queens, respectively; Kruskall-Wallis rank sum test: χ^2^ = 12.02, df = 2, *P* = 0.002).

**Figure 3:**
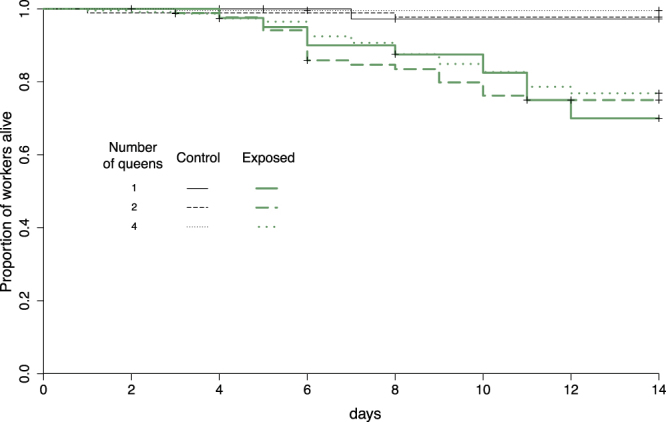
Survival of workers originating from control nests, in function of the number of founding queens (1, 2 or 4 queens) and exposure of workers to spores of *M. brunneum* (control versus exposed).

The fungal pathogen caused a significant mortality to the workers originating from control nests (Fig. 3; χ^2^ = 116.5, df = 1, *P* < 0.0001). The number of queens that initiated the colony did not influence worker survival overall (Fig. 3; χ^2^ = 1.6, df = 1, *P* = 0.2), nor the ability of workers to resist to the pathogen (Fig. 3; no significant interaction between queen number and pathogen exposure; χ^2^ = 2.2, df = 1, *P* = 0.14).

### Individual immune defences of queens

**Figure 4:**
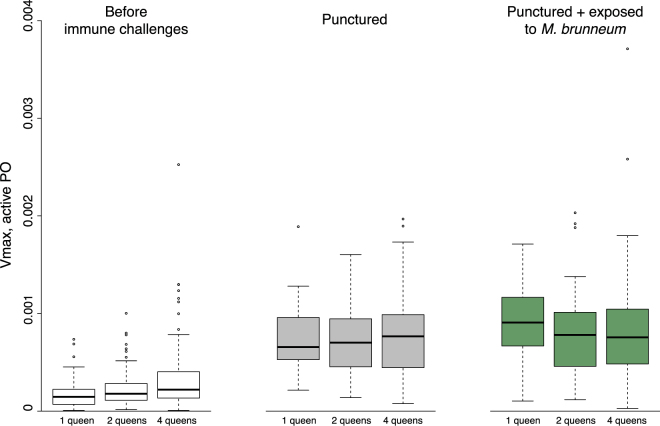
Level of active PO in queens, in function of the number of queens in founding associations and immune challenges. The boxplots show the median Vmax value, as well as the upper and lower quartiles. The whiskers encompass 1.5 times the interquartile range.

Before immune challenge, the level of active phenoloxidase (PO) in the queens’ hemolymph increased slightly but significantly with the number of founding queens (Fig. 4; ANOVA: *F* = 6.9, df = 2, *P* = 0.001). After cuticle puncture, the level of active PO increased greatly (Fig. 4; *F* = 445, df = 1, *P* < 0.0001), and did not vary with queen number (Fig. 4; *F* = 0.24, df = 2, *P* = 0.78). The level of active PO did not further change in response to exposure to fungal spores of *M*. *brunneum* (Fig. 4; *F* = 1.15, df = 1, *P* = 0.29).

**Figure 5:**
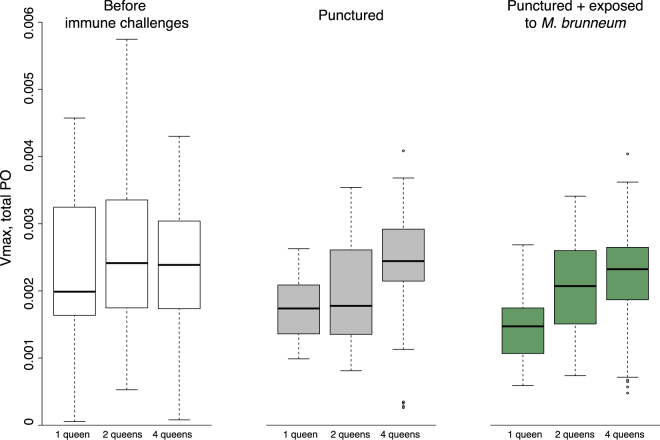
Level of total PO in queens, in function of the number of queens in founding associations and immune challenges. The boxplots show the median Vmax value, as well as the upper and lower quartiles. The whiskers encompass 1.5 times the interquartile range.

Before immune challenge, the level of total PO did not vary with queen number (Fig. 5; *F* = 0.47, df = 2, *P* = 0.63). After cuticle puncture, the total PO decreased significantly (Fig. 5; *F* = 17, df = 1, *P* < 0.0001), and increased with queen number (Fig. 5; *F* = 15.8, df = 2, *P* < 0.0001). The level of total PO did not further change in response to exposure to fungal spores of *M*. *brunneum* (Fig. 5; *F* = 0.79, df = 1, *P* = 0.38).

**Figure 6:**
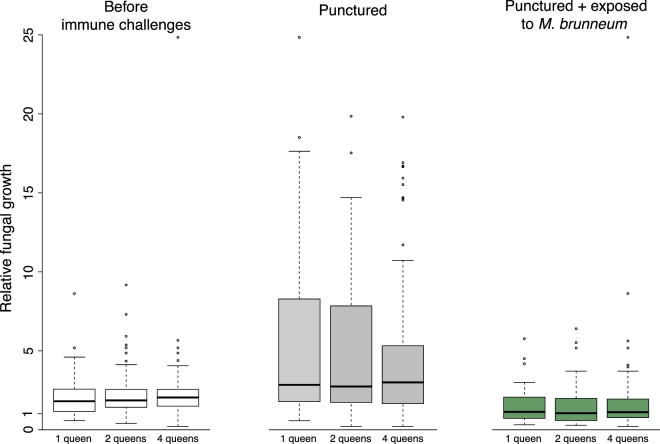
Growth of the fungus *M. brunneum* in a solution containing queen hemolymph, relative to growth in a hemolymph-free control solution, in function of the number of queens in founding associations and immune challenges. The boxplots show the median fungal growth value, as well as the upper and lower quartiles. The whiskers encompass 1.5 times the interquartile range.

Fungal growth in a solution containing queen hemolymph did not vary with queen number (Fig. 6; before challenges: *F* = 0.37, df = 2, *P* = 0.69; after puncture: *F* = 0.52, df = 2, *P* = 0.6). It increased significantly after cuticle puncture (Fig. 6; *F* = 55.5, df = 1, *P* < 0.0001) and tended to be reduced after queens’ exposure to the fungal pathogen (Fig. 6; *F* = 3.7, df = 1, *P* = 0.058).

## Discussion

We found no evidence that *L*. *niger* queens jointly establishing incipient colonies benefit from social immunity. Queens founding in associations did not engage in mutual grooming. Compared to solitary queens, they did not show higher resistance to the common soil fungal pathogen *M*. *brunneum*, did not decrease their investment in individual immunity, and their workers did not better resist disease.

This absence of social immunity among co-founding *L*. *niger* queens is in line with the results of Pull and colleagues^5^. In both experiments, queens performed almost no allo-grooming, even when exposed to fungal spores. In contrast, queens frequently self-groomed, particularly after having been in contact with fungal spores. A similar pattern has been documented for *F*. *selysi* workers: they increased self-grooming but did not perform more allo-grooming after being exposed to fungal spores^7^.

Queens that jointly founded incipient colonies did not benefit from improved disease resistance. When exposed to fungal spores, *L*. *niger* queens in groups did not have higher survival than solitary queens, both in our experiment and in the study by Pull and colleagues^5^. We used more stringent immune challenges, with cuticle puncture and group exposure by letting the ants walk on spores of another species of *Metarhizium*. This resulted in higher mortality of exposed queens, which showed no sign that they tolerate infection^5^. We also tested associations of four queens. Whatever the conditions, co-founding queens did not outperform lone foundresses in resisting *Metarhizium*, indicating that joint colony founding does not provide social immunity benefits to ant queens facing high doses of virulent generalist fungal pathogens.

Like their mothers, workers from incipient colonies founded by multiple queens did not show higher resistance to the fungal pathogen, compared to workers from colonies founded by one queen. Workers in colonies founded by multiple queens are expected to be genetically more diverse, because all queens in associations contribute to brood production^2,11^. It is possible that the benefits of genetic diversity have been offset by the social stress experienced by queens founding jointly, or that such benefits only occur with more specialized or less virulent pathogens. Yet, in the conditions tested, higher group genetic diversity did not translate into improved disease resistance.

Social immunity can result in a decreased investment in individual immunity^14^, and we found no evidence of such a decrease when measuring three components of the individual immune defence of queens. To the contrary, before the immune challenges queens in groups had higher phenoloxidase (PO) activity than solitary queens, as well as higher total PO after the challenges. Bumblebees kept in groups also increased active PO^22^. These results are consistent with an absence of social immunity, and suggest higher investment in individual immunity when in groups, possibly because of higher risks of pathogen transmission^20,23^.

Independently of queen number, the queens’ hemolymph tended to favour the growth of *M*. *brunneum*. The hyphae of this fungus penetrates the cuticle and grows inside the insect body, and may use hemolymph as food source^24^. In contrast, workers’ hemolymph has been shown to inhibit fungal growth^25^. It is possible that freshly mated queens are under energetic stress and are immunocompromised, maybe because of the constraints of sperm storage^3^. The growth of the fungus in hemolymph was higher after cuticle puncture and tended to be reduced when queens had been exposed to the fungus, which suggests that the antifungal defences of queens are modulated according to constraints and needs.

Joint colony founding conferred a clear demographic advantage. Incipient colonies founded by two and four *L*. *niger* queens produced 1.3 and 1.8 times more workers, respectively, than colonies founded by a single queen. Individually, each queen in co-founding associations invested less in reproduction, and thus saved resources for other uses, like individual immunity. Collectively, queens founding together produced a larger workforce. This pattern has been documented in several ant species, and reflects the fact that queens rely on their limited body reserves to rear the first cohort of workers^4,11,26^. A larger workforce is likely to confer a major advantage to incipient colonies when they start to compete with other colonies for foraging and brood raiding^4^.

In conclusion, in the tested conditions we found no evidence that joint colony founding confers social immunity benefits to ant queens. Co-founding queens did not engage in allo-grooming, but performed extensive self-grooming. Queens in group did not have higher resistance to the fungal pathogen than solitary queens, and did not produce more resistant workers. Finally, queens in groups increased their investment in some components of their individual immune defences. Queens fight to death after the first workers emerge, and the winner benefit from a larger workforce^4^. The absence of social immunity and elevated investment in individual immunity likely reflect the competitive nature of joint colony founding by ant queens.

## Material and Methods

### Queen sampling and experimental colony founding

The black garden ant *L*. *niger* is a common European species that nests in the soil. The species is strictly monogynous: all mature colonies are headed by one reproductive queen^2^. After the nuptial flight, queens shed their wings and are found by hundreds roaming on the ground, searching for a nest site. The queens initiate new colonies without assistance from workers (independent colony founding). Pleometrosis is facultative, with 18% of incipient colonies having multiple queens in a field population, and each pleometrotic nest containing two to five queens^2^. Queens in associations are unrelated. They do not forage and entirely rely on their energetic reserves to rear their first brood^26^. As soon as the first workers emerge, the queens engage in deadly fights, leaving only one queen alive^2,4^.

On July 12^th^, 2013, we collected young mated queens that were walking on the campus of the University of Lausanne after the nuptial flight. The next day, we placed the queens in experimental nests either alone, in pairs, or in groups of four (N = 45 replicates for each queen number category). Experimental nests consisted of test tubes (17.5 cm long, 1.5 cm diameter) with water blocked by cotton wool at the bottom.

### Queen behaviour, immune challenges and survival

We monitored queen behaviour by scanning each nest for five seconds, five times per day, over seven days. We recorded instances of self-grooming, allo-grooming and trophallaxis (oral exchange of liquid). After 10 days, all queens were subjected to a first immune challenge, which consisted in a small puncture of the thorax with a glass micro capillary and the extraction of 1 µl of hemolymph (that we used to measure individual immunity, see below). At day 22, queens from 22 out of the 45 experimental nests in each queen number category, respectively, were exposed to the generalist entomopathogen *Metarhizium brunneum* ^27^. The genus *Metarhizium* is widely distributed and common in Switzerland^28^, yet information on strains and dose in nature is still lacking. In exposed nests, 500 µl of spore solution (1.75 × 10^8^ spores/ml in 0.05% Tween 20) were deposited on a filter paper (6.5 × 2 cm). In control nests, queens were exposed to 500 µl of 0.05% Tween 20. After this fungal challenge, we monitored again the queen behaviour over seven days, as described above. Two days after the fungal challenge, which is enough time for spores to germinate and elicit an immune response, all the queens, in both control and fungus-exposed nests, were subjected to a second cuticle puncture in order to extract hemolymph. We monitored queen survival for a total of 87 days, and counted the number of workers produced in each nest with queens alive at day 81.

### Worker resistance

We tested if the resistance of workers to *M*. *brunneum* depended on the number of queens that founded their nest. We used workers from control nests that had no previous exposure to the pathogen. From each control nest that produced at least 10 workers, we made as many groups of five workers as possible (2 to 10). These five-worker groups were kept in 9 cm diameter petri dishes with a filter paper disk at the bottom. Half of the groups from each nest were exposed to spores of *M*. *brunneum* (500 µl of 0.05% Tween 20 with 1.8 × 10^8^ spores/ml deposited on the filter paper), while the other half of the groups were kept as control (500 µl of spore-free 0.05% Tween 20 deposited on the filter paper). We monitored worker survival over 14 days.

### Immune measures

The individual immunity of queens was assessed by measuring active phenoloxidase (PO), total PO and fungal growth in their hemolymph. Active PO is an essential component of the innate immune defence of insects. This enzyme is involved in the melanization of pathogens and of damaged tissues. Prophenoloxidase is converted in active PO when particles of microbial origin are present, or after wounding^29,30^. We measured active PO and total PO (=active PO + prophenoloxidase), following the methods described in Castella *et al*.^31^. Briefly, the sample of 1 µl of hemolymph was diluted in 10 µl of sodium cacodylate, and 3 µl of diluted hemolymph was used per measure of PO^31^. The absorbance was measured at 492 nm every 10 s for 800 reads at 30 °C. We analysed the active PO and total PO curves with the software PO-CALC^32^.

Fungal growth in queen’s hemolymph was measured as described in Konrad *et al*.^25^. We used 96-well plates containing 2 µl of fungal spore solution (8 × 10^6^ spores/ml in 0.05% Tween 20) diluted in 50 µl of Sabouraud Dextrose Broth (SDB). We added either 3 µl of diluted hemolymph or 3 µl of sodium cacodylate as hemolymph-free controls. Fungal growth was estimated by subtracting the absorbance in a spectrophotometer immediately after the set up from the absorbance after 24 hours^25^. Fungal growth in hemolymph was standardized with respect to controls: i.e., fungal growth in wells with hemolymph was divided by average fungal growth in hemolymph-free controls.

### Statistical analyses

For each queen, we calculated the proportion of occurrence of self-grooming (number of observations divided by number of scans when the queen was alive). To determine if the occurrence of self-grooming depended on the number of queens in founding associations and on the immune challenges, we used two mixed-effects models. The proportion of self-grooming events was the response variable. In the first model, queen number and cuticle puncture were included as explanatory variables, and the nest and queen identity as random factors (to compare the proportion of self-grooming by the same queens before and after puncture). In the second model, queen number and exposure to fungal spores were included as explanatory variables, and the nest as random factor. The proportion of self-grooming was square-root transformed to satisfy the assumptions of normality of residuals and homogeneity of variances.

Queen survival was analysed with a Cox proportional hazards model. The proportion of queens alive was the response variable. The explanatory variables were the number of queens in founding associations, the exposure to fungal spores, and the interaction between the two factors (we expect an interaction if queens in groups are more resistant to the pathogen). The experimental nest was included as a random factor. Worker survival was analysed in a similar manner, with the group of workers nested in the nest of origin as random factors. We used Cox mixed-effects models, as implemented in the package “coxme”^33^.

We analysed if queen number, cuticle puncture and exposure to fungal spores influenced the levels of active PO, total PO and fungal growth in hemolymph with mixed effects models. We analysed each component of immunity separately, using the level of active PO, total PO or fungal growth in hemolymph as response variable in each model. We constructed three models to analyse i) immune activity before immune challenges, using queen number as explanatory variable and the nest as random factor; ii) change in immune activity after cuticle puncture, using cuticle puncture as explanatory variable, as well as the nest and queen identity as random factors (to compare immune measures from the same queens before and after puncture) and iii) immune activity after exposure to fungal spores, using queen number and exposure to fungal spores as explanatory variables and the nest as random factor. Active PO was squared-root transformed and fungal growth in hemolymph log transformed to satisfy the assumptions of normality of residuals and homogeneity of variances.

### Data availability

The datasets generated during and/or analysed during the current study are available in the Dryad repository.
